# Distinct genetic patterns and natural history of *OPA1*-related auditory neuropathy in Chinese population

**DOI:** 10.1186/s13023-025-04040-4

**Published:** 2025-10-17

**Authors:** Hongyang Wang, Tao Shi, Wenjia Wang, Jin Li, Ziyi Chen, Lan Lan, Linyi Xie, Fen Xiong, Dayong Wang, Jing Guan, Qiuju Wang

**Affiliations:** 1 Senior Department of Otolaryngology Head and Neck Surgery, The 6th Medical Center of Chinese PLA General Hospital, Chinese PLA Medical School, Beijing, 100853 China; 2 State Key Laboratory of Hearing and Balance Science, Beijing, 100853 China; 3 National Clinical Research Center for Otolaryngologic Diseases, Beijing, 100853 China; 4 Key Laboratory of Hearing Science, Ministry of Education, Beijing, 100853 China

**Keywords:** *OPA1*, Auditory neuropathy, De novo, Dominant optic atrophy plus

## Abstract

**Background:**

Auditory neuropathy (AN) represents a clinical manifestation of *OPA1*-related diseases. The diagnostic process of these diseases is challenging owing to the broad spectrum of intermediate phenotypes and diverse inherited patterns. The aim of this study was to comprehensively delineate the feature of *OPA1*-related patients in a Chinese AN cohort, encompassing the incident rate, inherited pattern, detailed audiological characteristics, and genotype-phenotype correlation.

**Methods:**

Between 2003 and 2020, 452 unrelated probands with a diagnosis of AN were referred to our laboratory for molecular genetic investigation with high-throughput sequencing. Sanger sequencing was performed on the probands and their parents to verify the genetic results. Patients diagnosed as AN by clinical evaluation, auditory brainstem responses, otoacoustic emission and/or cochlear microphonic. Comprehensive auditory evaluations were conducted on *OPA1*-related patients, and some of them were performed a follow-up study.

**Results:**

We identified seven probands (1.55%, 7/452) with *OPA1* variants in seven unrelated families, demonstrating distinct genetic patterns, including one family with rare autosomal recessive (AR) inheritance, six families with autosomal dominant (AD) inheritance (three were AD de novo). The AN phenotype was observed in all patients prior to the second decade of life, with AN serving as the initial presenting symptom in two patients. Additionally, probands with the rare AR inheritance exhibited a more severe phenotype. A total of eight *OPA1* variants were identified, including a novel variant c.2013 + 5G > C. The GTPase domain of *OPA1* exclusively harbored missense variants, and 85.71% (6/7) of the patients carried one of missense variants in *OPA1*. The observed phenotypes exhibited a broad spectrum of manifestations, encompassing vestibular dysfunction and developmental delay, with varying degrees of hearing loss. Among the seven patients, four exhibited severe to profound hearing loss. The annual rates of hearing loss at the frequencies of speech were 2.74 dB/year for one patient, who underwent a 10-year-old follow-up.

**Conclusion:**

Our results indicated the distinct genetic patterns and variable phenotypic characteristics of *OPA1*-related AN in the Chinese population. The audiological features of *OPA1*-related patients were comprehensively described as exhibiting AN. We identified one novel splicing variants that expand the genetic spectrum of *OPA1* variants in AN.

**Supplementary Information:**

The online version contains supplementary material available at 10.1186/s13023-025-04040-4.

## Background

Auditory neuropathy (AN) refers to an auditory information processing disorder caused by dysfunction of the inner hair cell (IHC), synapses, spiral ganglion neuron (SGN), and/or the auditory nerve itself [1]. Patients with AN character absent or abnormal response in auditory brainstem responses (ABR) and the presence of cochlear microphonics (CM) and/or otoacoustic emission (OAE), revealing normal function of outer hair cell (OHC) and abnormal function of auditory pathway [2]. The etiology of auditory neuropathy is multifactorial, with approximately 40% of cases being attributed to underlying genetic disorders [3, 4]. The diversity of lesion sites and the complexity of etiology contribute to the possibility of AN occurring in association with other syndromes. *OPA1* is one of the gene that association with AN and other system disorder [5].

*OPA1*, located on chromosome 3q28-q29, encodes a mitochondrial dynamin-related GTPase in the intermembrane space [6]. *OPA1* variants contributed to genetic diseases in distinct genetic patterns, including autosomal dominant optic atrophy plus (DOA+) (OMIM: #125250) following an autosomal dominant (AD) pattern, and Behr syndrome (OMIM: #210000) following an autosomal recessive (AR) pattern. The occurrence of biallelic *OPA1* variants in patients may be relatively rare, leading to a more severe manifestation of the disease [7–10]. To our knowledge, the current available information suggested that a total of ~ 25 patients with biallelic *OPA1* variants have been reported globally [11].

Both of DOA + and Behr syndrome can exhibit hearing loss. The most prevalent extra-ocular manifestation in DOA + is sensorineural hearing loss, which is observed in nearly two-thirds of DOA + patients [12]. The gradual deepening understanding of deafness has led to the proposal that AN serves as a neurological disorder, representing the pathophysiological mechanism underlying hearing impairment in patients with DOA+ [13–15]. Therefore, it is important to note that simple audiological tests such as pure tone audiometry are insufficient for diagnosing AN. Due to the possibility of some patients with AN having hearing threshold within the normal range, this can underestimate the severity of hearing impairment in *OPA1*-related patients. Behr syndrome characterized by much more severe neurological manifestations including optic neuropathy (OA), hearing loss, peripheral neuropathy, and retarded development. However, it remains uncertain whether AN is a contributing factor to hearing loss in this particular disease, and rare studies have provided detailed descriptions and nature history of the auditory function of *OPA1*-related patients. In addition, the implementation of precise early molecular diagnosis further enables effective intervention and treatment [16–18].

Here, we performed a retrospective study of 452 patients with a clinical diagnosis of AN and identified a total of seven probands with *OPA1* variants as detected by genome sequencing from seven unrelated families, presenting three distinct genetic patterns: AD, AD de novo, and rare AR (2.25%, 7/452). A total of eight different *OPA1* variants have been identified, and we conducted comprehensive audiological testing to characterize the hearing dysfunction in these probands.

## Methods

### Subjects collection

This was a retrospective cohort study of patients, and a total of 452 cases with a clinical diagnosis of AN by the Institute of Otolaryngology, Chinese PLA General Hospital between January 2003 and December 2020. The medical records and samples of all patients were used for research purposes with their informed written consent. In the case of underage probands, informed written consent was obtained from their parents or guardians. This study was approved by the Committee of Medical Ethics of Chinese PLA General Hospital (S2020‑228‑01). Written informed consent was obtained from participants. The data pertaining to patients from the Multicenter Study on Clinical Diagnosis and Intervention of Acoustic Neuropathy (registration number: ChiCTR2100050125; date of registration: August 16, 2021; institution hospital: Chinese PLA General Hospital).

### Clinical evaluations

The diagnostic criteria of AN were based on the Chinese clinical practice guideline of auditory neuropathy (version 2022) [19]: The ABR is indicative of absence or abnormality, while the CM and/or OAE results remain within normal range. Medical evidence of hearing loss and other clinical abnormalities was identified in both affected and unaffected family members. The auditory status was assessed through the implementation of pure tone audiometry (PTA), speech discrimination score (SDS), ABR, OAE, Electrocochleogram (ECochG), and cervical vestibular evoked myogenic potentials (cVEMP). The temporal bone was examined using high-resolution computed tomography (CT) scans, while the internal auditory canal was assessed through magnetic resonance imaging (MRI), in order to rule out any potential neuropathic or anatomical disorders. Ophthalmic auxiliary examination included funduscopy, optical coherence tomography (OCT), pattern electroretinogram (PERG), and flash visual evoked potential (FVEP). Electromyography is used to detect muscle nerve conduction, including sensory nerve action potential (SNAP) and motor nerve conduction velocity (MCV).

### Genetic techniques

A total of 452 patients with AN were enrolled in the study. All patients underwent high-throughput sequencing, with 233 patients receiving whole-genome sequencing (WGS) focused on AN-related gene, and 219 patients undergoing targeted gene capture sequencing for known hereditary hearing loss genes (HHL Panel) [20]. Following genetic tests, seven probands were identified as carrying at least one *OPA1* variant. Sanger sequencing was conducted on both the probands and their family members to validate any identified gene variants, following previously established protocols. The assessment of pathogenicity in relation to genetic variants was conducted according to the standards and guidelines set forth by the American College of Medical Genetics and Genomics/ Association for Molecular Pathology (ACMG/AMP) [21]. For In Silico variant deleteriousness predictions, we analyzed the variants in the Variant Effect Predictor on-line website tools (Ensembl, https://grch37.ensembl.org/Multi/Tools/VEP) [22].

## Results

### General clinical information of OPA1-related patients

**Fig. 1:**
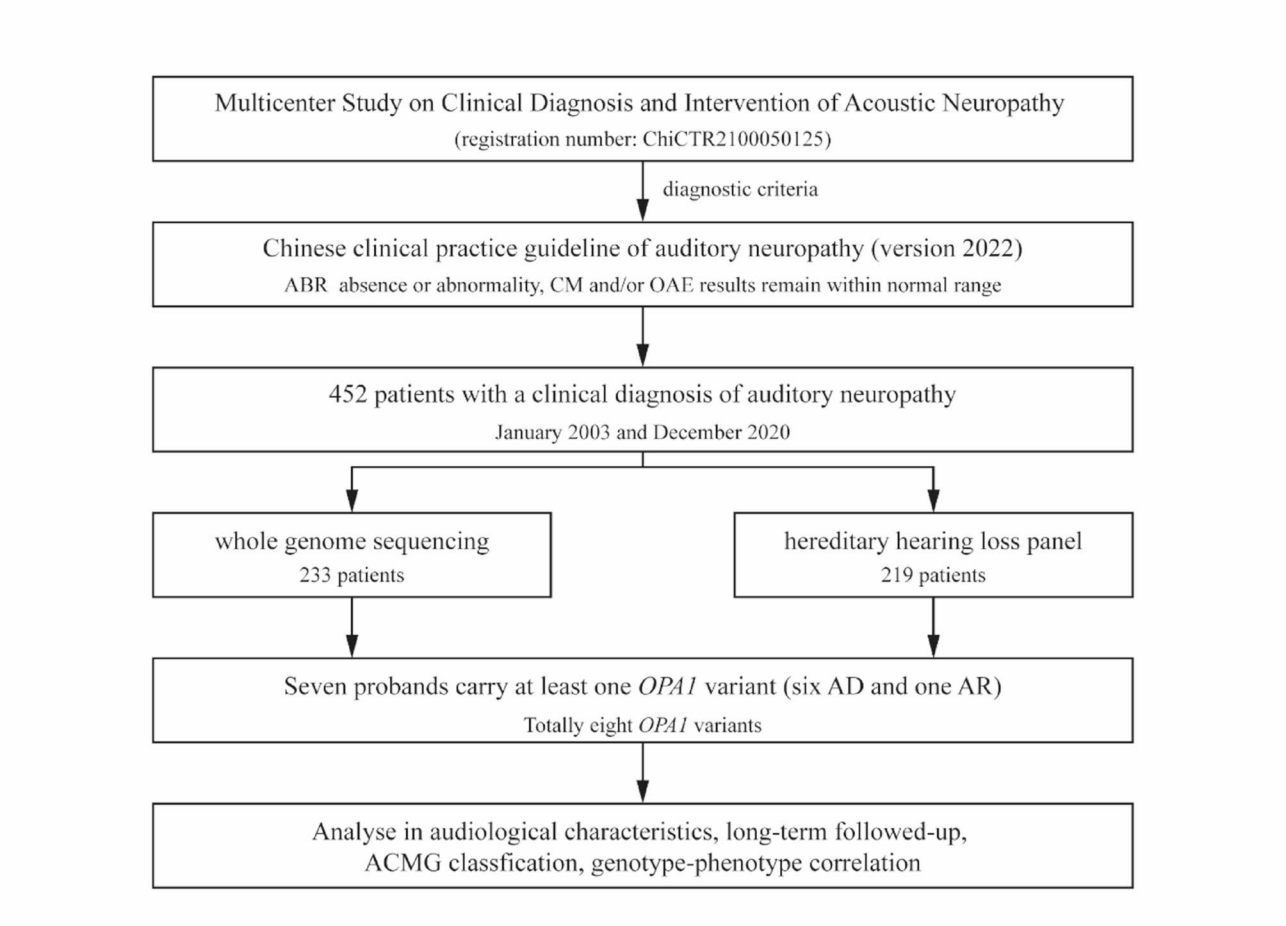
Flowchart depicting the inclusion criteria for *OPA1*-related patients

According to the flowchart shown in Fig. 1, we screened out 7 *OPA1*-related AN probands from 452 patients clinically diagnosed with AN. We hereby presented the clinical histories of seven probands from seven unrelated Chinese families and a summary of the clinical and auditory findings is reported in Table 1. The seven *OPA1*-related probands comprised of three females and four males, with the age range of test from 20 months to 24 years (with a median age of 7 years) and the age range of onset from 0 years to 10 years (with a median age of 2 years). The onset age of AN was before the second decades of life, with two probands presenting hearing loss as the initial symptom while the remaining individuals exhibited vision impairment as the primary symptom.

**Table 1:** Clinical and audiological data from the *OPA1*-related patients

Family ID	1006835	1507337	1707789	1707823	1808147	1908306	Y2953973
Clinical							
Gender	Female	Female	Male	Male	Male	Male	Female
Age test	24Y	7Y	8Y	6Y	3Y	21Y	20 M
Age onset	9-10Y	2Y	5 M	0Y	2Y	childhood	3 M
Sign at onset	Hearing	Vision	Vision	Hearing	Vision	Vision	Vision
Deaf onset	9–10	6Y	8Y	0Y	2Y	Childhood	1Y
Tinnitus	-	-	-	-	-	+	-
Auditory neuropathy	Yes	Yes	Yes	Yes	Yes	Yes	Yes
Optic atrophy	Yes	Yes	Yes	NA	Yes	Yes	Yes
Nystagmus	-	-	Yes	-	-	-	Yes
Gait instability	-	-	Yes	-	Yes	-	Cannot walk
RD	-	-	-	-	-	-	Yes
Other impairment	-	-	-	Jaundice	Trichiasis	-	Strabismus
Genotype							
Allele 1	c.1333 C > G	c.1555G > A	c.805T > C	c.2635_2639 del	c.1208T > C	c.1201G > C	c.356T > C
Allele 2	-	-	-	-	-	-	c.2013 + 5G > C
Audiology (Left/Right)							
Hearing	Both severe	Both severe	Both profound	NA	Both profound	Moderate/mild	Both moderate
PTA (dB HL)	67.5/70	65/77.5	88.75/81.25	NA	87.5/83.75	38.75/27.5	37.75/37.75
SDS(%)	28/24	0/20	NA	NA	NA	20/24	NA
Tympanogram	As/A	A/As	As/As	NA	As/A	A/A	As/As
Stapedial reflex	ABS/ABS	ABS/ABS	ABS/ABS	NA	ABS/ABS	ABS/ABS	ABS/ABS
DPOAE	+/+	+/+	+/+	ABS/ABS	+/+	+/+	+/+
ABR-V (ms)	ABS/ABS	ABS/ABS	ABS/ABS	ABS/ABS	ABS/ABS	ABS/ABS	6.63/6.58
CM	+/+	+/+	+/+	+/-	+/+	+/+	NA
ECochG: -SP/AP	NA	0.84/1.87	NA	NA	NA	AP absent	NA
Follow-up							
Follow-up age (M)	143	120	NA	NA	NA	39	NA
Type of intervention	HA (L)	None	NA	NA	NA	HA (L)	NA
Age intervention (Y)	27	None	NA	NA	NA	4	NA
CoAP	5	6	NA	NA	NA	5	NA
SIR	4	5	NA	NA	NA	5	NA

Note: *OPA1* (NM_015560.3); ABS (absent); CM (cochlear microphonic); CoAP (categories of auditory performance); M (months); NA (not applicable); Y (years); PTA (pure-tone average); RD (retarded development); SDS (speech discrimination score); SIR (speech intelligibility rating); HA (hearing aids)

### Audiological phenotype characteristics of OPA1-related patients

**Fig. 2:**
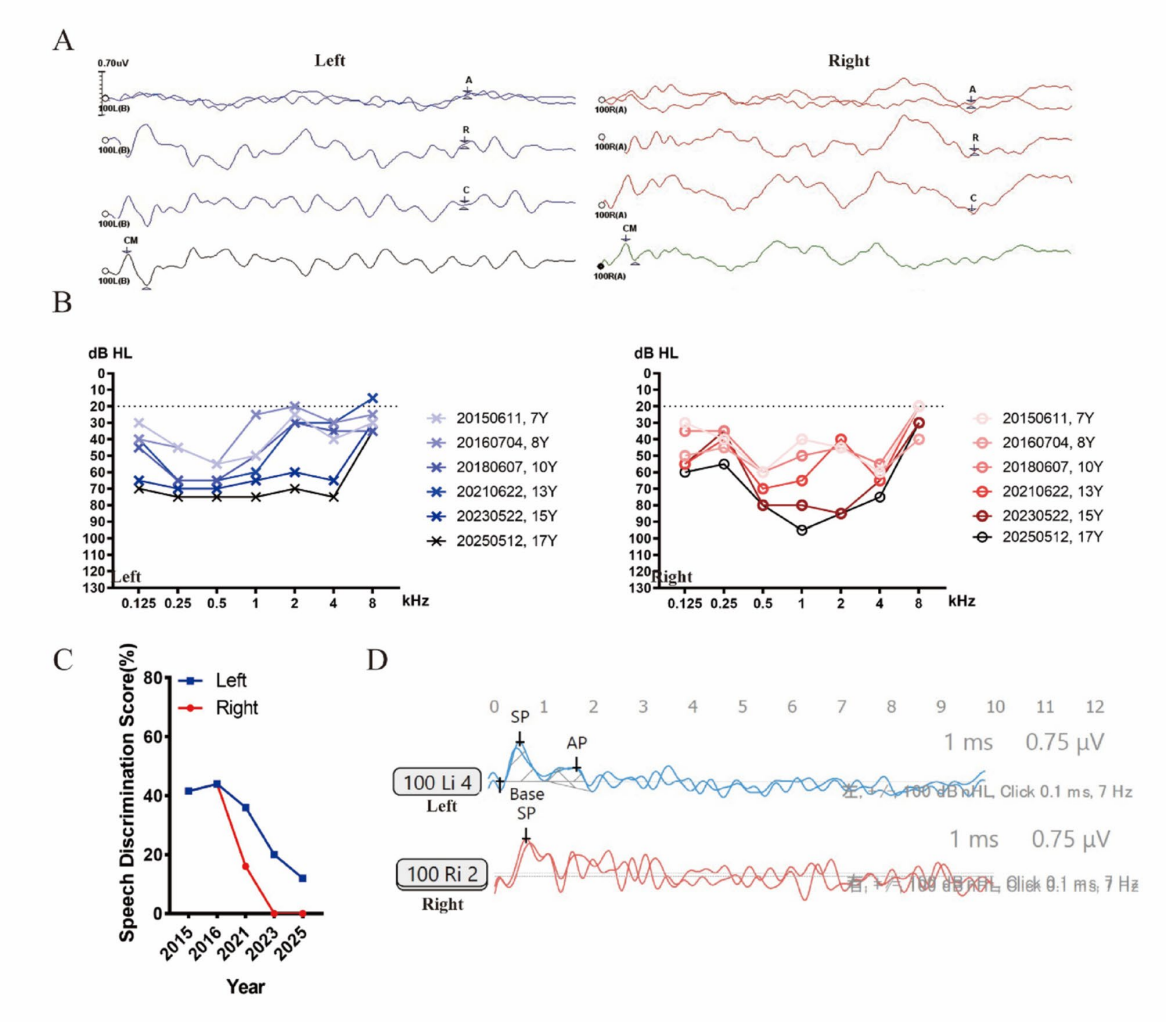
The representative audiology phenotype of *OPA1*-related patients. These results were conducted from the proband of Family1507337. (A) Absent ABR waves at 100 dB nHL stimulus in both ears. CM waves can be recorded in both ears. (B) Audiograms of the proband from 2015 to 2025 displayed moderate to profound sensorineural hearing loss (Y means years). (C) SDS results from 2015 to 2025 showing decreased SDS. (D) ECochG shows the -SP/AP were larger than 0.4 in left ear, while absent AP wave in the right ear. Blue, left ear; red, right ear. ABR, auditory brainstem response; CM, cochlear microphonic; SDS, speech discrimination score

The manifestations of AN were observed in all probands, along with typical audiological findings as depicted (Fig. 2A-D, proband from Family 1507337). ABR was abnormal or delayed responses latency of wave V, while CM could be recorded and/or DPOAE could be elicited at most frequencies. The proband II.1 from Family 1707823 did not record DPOAE in either ear, but his left ear can record CM. All probands exhibited binaural hearing loss and hearing thresholds were varied from mild to profound, with four out of seven probands showing severe to profound. Tympanograms showed “A” type or “As” type, indicating no abnormal middle ear function, while the acoustic middle ear reflex were absent at all frequencies in all probands. The SDS was severely impaired beyond what would be expected based on the degree of hearing loss. Among three probands tested, it was observed that SDS were consistently below 30%. Notably, one proband exhibited a score of 0% in her left ear during the most recent test. The EcochG was conducted in two probands, one of whom had a significantly higher -SP/AP value (0.84 for left ear, 1.87 for right ear), while the other proband was unable to record the AP wave in both ears.

A total of 4 probands conducted temporal CT, and no abnormalities were observed in 2 probands, except for proband II.2 from Family 1507337, who exhibited high signal intensity in the bilateral jugular fossa and a group of paranasal sinusitis. The proband II.2 from Family 1707798 presented with right maxillary sinusitis. Furthermore, a total of 4 probands conducted MRI examination, and no abnormalities were observed in 2 probands, except for proband II.2 from Family 1507337 displayed bilateral cochlear nerve hypoplasia, while proband II.2 from Family 1707798 revealed a distinct vascular shadow in the left cerebellopontine angle that closely approached the left facial nerve and vestibular nerve.

### Natural history and follow up of OPA1-related patients

The proband II.2 from Family 1507337 was longitudinally followed for a duration of 10 years (2015–2025), during which the proband displayed progressive bilateral hearing loss (Fig. 2B). From the ages of 7 to 17 years, the degree of hearing loss in the proband increased from moderate to profound. The speech-frequency (0.5–4 kHz), low-frequency (0.125–0.5 kHz), mid-frequency (1–2 kHz), and high-frequency (4–8 kHz) hearing decreased at 3.18 dB/year (range, 46.88–78.63 dB HL), 2.59 dB/year (range, 43.33–69.15 dB HL), 4.13 dB/year (range, 40.00–81.25 dB HL), and 1.63 dB/year (range, 37.50–53.75 dB HL), respectively. Between 2015 and 2025, SDS results continued to decrease until it was 0% in the right ear and 12% in the left ear (Fig. 2C). She had undergone three EcochG tests. The first test conducted in 2016, only the right ear recorded -SP and AP, with absolute values of -SP/AP was 1.53. In 2021, she showed the -SP wave only, without an obvious AP wave in the right ear, and an absolute values of ‐SP/AP value 2.89 in left ear (Fig. 2D). In 2023, the absolute values of ‐SP/AP were 0.84 and 1.87 in the left ear and right ear, respectively. The proband underwent three cVEMP tests over a span of six years. Two of these tests resulted in unilateral or bilateral absence of cVEMP. Additional vestibular-function testing showed normal oculomotor function with no significant nystagmus, as well as normal vestibular caloric assessment, but reduced bilateral horizontal semicircular canal function.

The successful follow-up of 3 *OPA1*-related probands was conducted, including Family 1006835, 1507337, and 1908306. The follow-up period was 143, 120, and 39 months, respectively. The average points of categories of auditory performance (CoAP) and Speech intelligibility rating (SIR) scores were 5.33 and 4.67, respectively (Table 1).

### Extra-ear phenotype of OPA1-related patients

**Fig. 3:**
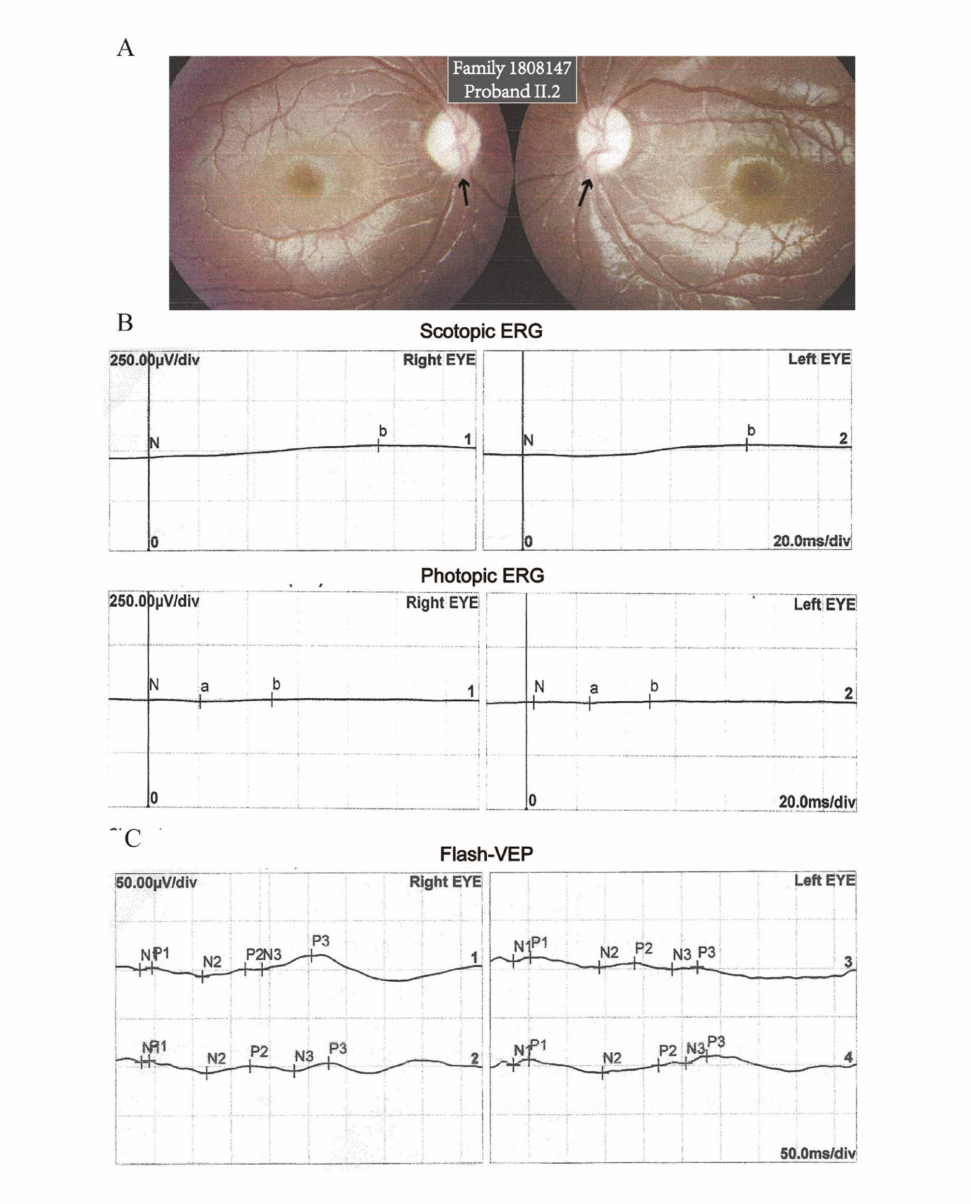
The representative visual phenotype of *OPA1*-related patients. (A) Fundus photographs of the proband of Family 1808147. Pallor of the optic disc was present in this case. (B) The amplitude severity of both eyes was reduced during ERG testing under conditions of light and dark adaptive stimulation. (C) The Flash-VEP results revealed prolonged P2 latency and decreased amplitude in both eyes

The spectrum of visual impairment phenotypes in probands was extensive. The 6-year-old proband from Family 1707823 did not present any visual abnormalities, at least during the time of the examination. In addition to the aforementioned proband, the occurrence of OA was observed in all the six *OPA1*-related probands. However, three exclusively exhibited OA as a visual abnormality. The typical visual findings observed through flash-VEP and ERG examinations revealed optic nerve conduction blockade, as well as dysfunction in cone and rod cells (Fig. 3A-C). The remaining probands included two cases of nystagmus, one case of strabismus, and one case of trichiasis.

In addition to vision and auditory impairments, some probands exhibited gait instability. The proband from Family 1707798 exhibited decreased sural nerve SNAP amplitude and conduction velocity on the right side, as well as decreased left median nerve SNAP amplitude by electromyography. Furthermore, there was a slowed decrease in MCV of the left median nerve.

### Genotype analysis of OPA1 in auditory neuropathy patients

**Fig. 4:**
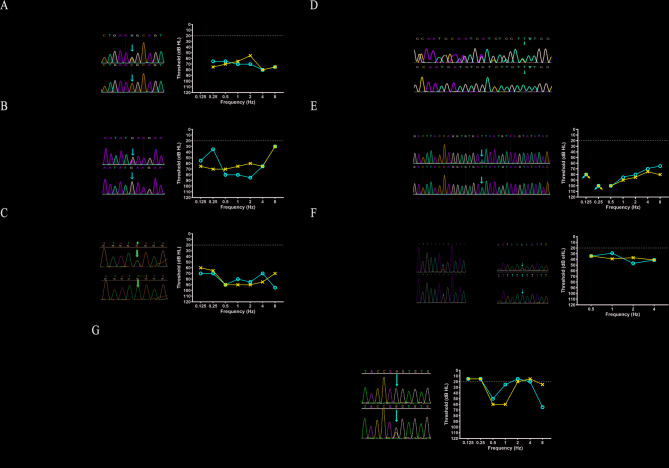
Complete family pedigree of *OPA1-*related patients. (A) Family 1006835. The proband II.2 was a 24-year-old woman who was noted for hearing loss at 9–10 years old. At the age of 17, a hearing aid was fitted to the right ear with satisfactory outcomes. Her bother II.3, a 21-year-old man, experienced the onset of hearing loss at age 14 and visual disturbance at age 16. (B) Family 1507337. The proband II.1, a 7-year-old girl, initially presented with visual deterioration at the age of 2 years and diagnosed with optic atrophy (OA). (C) Family 1707798. The proband II.1, a 4-year-old boy, presented with nystagmus at the age of 5 months and subsequently made repeated visits due to ongoing visual impairment. Following head trauma five months ago, he developed binaural hearing loss and communication difficulties. In his daily life, he exhibited symptoms of exercise intolerance and gait instability. (D) Family 1707823. The proband II.1 was a 6-year-old boy. The newborn’s primary hearing screening and secondary hearing screening showed that he passed the left ear, but failed the right ear. Currently, he exhibited communication challenge and delayed psychomotor development. (E) Family 1808147. The proband II.2, a 3-year-old boy, presented with hearing loss and exhibited speech characterized by unclear pronunciation at the age of 2.5 years. He experienced foot pain following a significant amount of physical activity throughout the day, and exhibited an unsteady and rolling gait on usual activities. Moreover, the proband exhibit abnormal vision function at age of 2 years and diagnosed with binocular OA. (F) Family Y2953973. The proband II.2, a 1-year-old girl, presented with nystagmus and difficulties in maintaining fixation on moving objects at the age of 3 months. She diagnosed with binocular optic atrophy at the age of 4 months. Currently, she exhibited communication challenge and was accompanied by psychomotor retardation at age of 15 months. (G) Family 1908306. This 22-year-old proband belongs to a Chinese family with at least other five affected individuals with autosomal-dominant transmission of AN and OA. He has suffered a progressive hearing loss and visual impairment since childhood. Furthermore, he exhibited poor comprehension and severe tinnitus

In the population studied, at least an *OPA1* variants (NM_015560.3) was found in seven affected probands from seven unrelated families (Fig. 4). More specifically, seven families had distinct genetic pattens, including AR (Family Y2953973), AD (Family 1006835, 1707823, and 1908306), and AD de novo (Family 1507337, 1707789, and 1808147). The parental samples were subjected to analysis, and paternity testing was conducted in all of these families to confirm the presence of these de novo variants. The proband of Family Y2953973 with AR genetic pattens exhibited a more severe phenotype compared to those with AD genetic pattens, including symptoms such as nystagmus, development delay, inability to ambulate, strabismus.

**Fig. 5:**
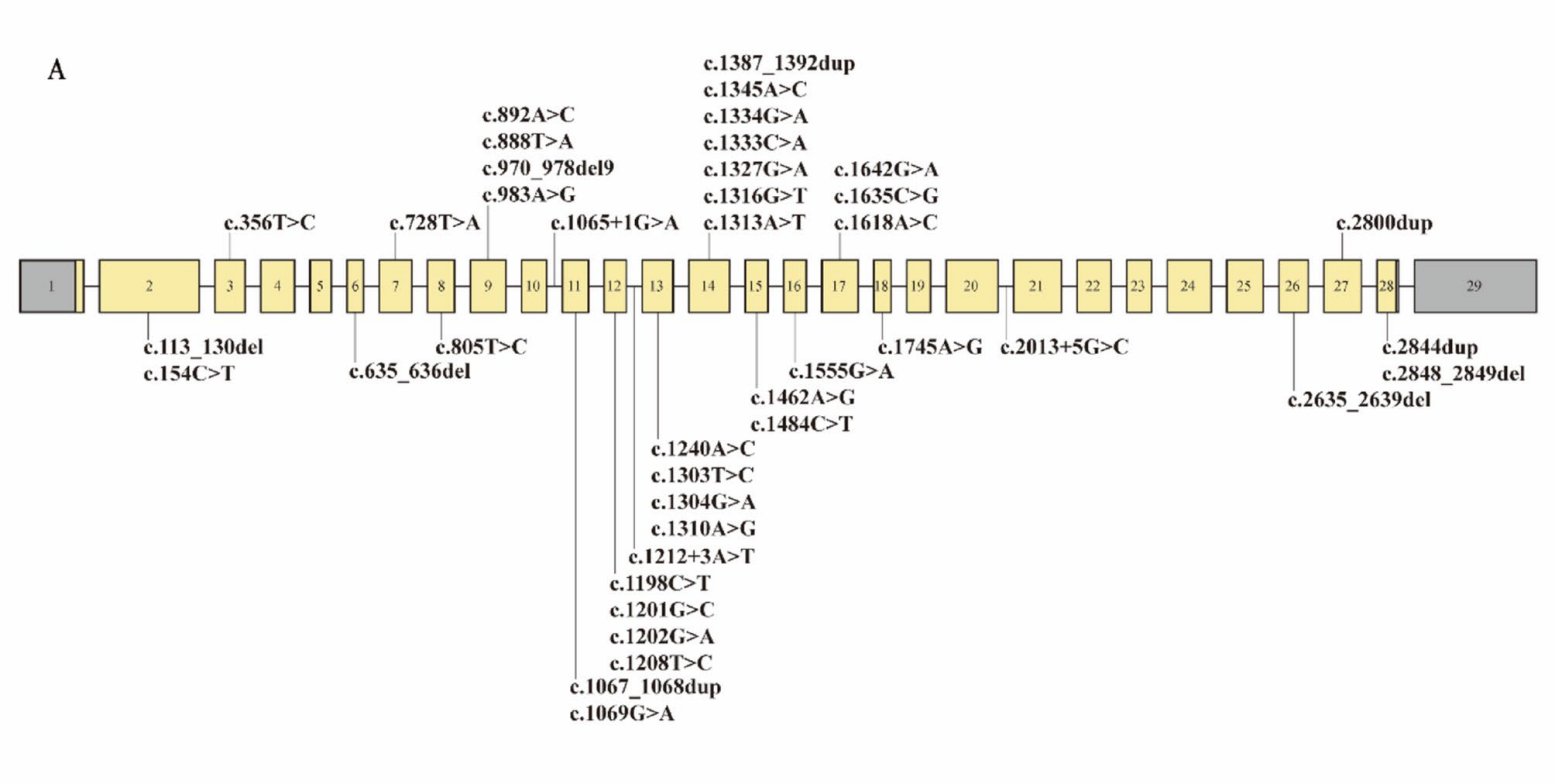
Summary of reported hearing loss-related variants in *OPA1*. (A) *OPA1* (NM_015560.3) consisted of 29 exons. The gray shading represents noncoding segments. A total of 41 variants were summarized

We have identified a total of eight *OPA1* variants, including three reported *OPA1* variants: c.356T >C, c.1201G >C, and c.1208T >C; four reported *OPA1* variants by our group [23, 24]: c.805T >C, c.1333 C >G, c.1555G >A, and c.2635_2639del; a novel *OPA1* variants: c.2013 + 5G >C. According to the ACMG/AMP guidelines, out of the 8 variants, 5 were classified as likely pathogenic and 3 as pathogenic (Table 2). These *OPA1* variants included 6 missense variants, 1 splicing variant, and 1 frameshift variants. Most of probands (6/7, 85.71%) with AN carried one of the missense variants in the *OPA1*. The results of In Silico variant deleteriousness predictions showed that all missense and splicing variants would cause damage to protein function (Table 3). We systematically summarized the reported pathogenic *OPA1* variants associated with hearing loss, totaling 41 variants, including the 8 variants identified in this study (Fig. 5 and Table S1). Notably, 27 of these variants (65.85%) were located within the GTPase domain (Codons 285–561).

**Table 2:** *OPA1* variations identified

Number	Family ID	Nucleotide change	Amino acid change	Pathogenicity evidence	Pathogenicity (ACMG)
1	Y2953973	c.356T > C	p.Phe119Ser	PM2 + PM3 + PP3 + PP4	LP
2	1707789	c.805T > C	p.Ser269Pro	PS2 + PM2 + PP3 + PP4	LP
3	1908306	c.1201G > C	p.Gly401Arg	PP1-strong + PM1 + PM2 + PM5 + PP3 + PP4	P
4	1808147	c.1208T > C	p.Ile403Thr	PS2 + PM1 + PM2 + PP3 + PP4	P
5	1006835	c.1333 C > G	p.Arg445Gly	PM1 + PM2 + PM5 + PP1-supporting + PP3 + PP4	LP
6	1507337	c.1555G > A	p.Glu519Lys	PS2 + PM1 + PM2 + PP3 + PP4	P
7	Y2953973	c.2013 + 5G > C	-	PS1_moderate + PM2 + PP3 + PP4	LP
8	1707823	c.2635_2639del	p.Phe880Alafs*21	PVS1 + PM2	LP

Note: *OPA1* (NM_015560.3)

**Table 3:** In Silico variant deleteriousness predictions

Nucleotide change	SIFT	REVEL	AlphaMissense	SpliceAI				MaxEntScan	
				DS_ AG	DS_ AL	DS_ DG	DS_ DL	Alt	Ref
c.356T > C	Deleterious(0)	0.622	0.913	-	-	-	-	-	-
c.805T > C	Deleterious(0)	0.943	0.999	-	-	-	-	-	-
c.1201G > C	Deleterious(0)	0.986	1.0	-	-	-	-	-	-
c.1208T > C	Deleterious(0)	0.867	0.994	-	-	-	-	-	-
c.1333 C > G	Deleterious(0)	0.887	0.986	-	-	-	-	-	-
c.1555G > A	Deleterious(0)	0.937	0.997	-	-	-	-	-	-
c.2013 + 5G > C	-	-	-	0	0	0.47	0.81	-1.571	4.364

Note: *OPA1* (NM_015560.3); DS: Δscore; AG: acceptor gain; AL: acceptor loss; DG: donor gain; DL: donor loss Alt: alternate sequence score; Ref: reference sequence score; The threshold for impact: SIFT < 0.05; REVEL > 0.5; AlphaMissense > 0.564 SpliceAI Δscore > 0.2; MaxEntScan: diff = Ref-Alt > 0 and Alt < 6.2, using ENIGMA thresholds (https://enigmaconsortium.org/)

## Discussion

In this study, we found seven probands with *OPA1* variants from seven unrelated families in a Chinese AN cohort (1.55%, 7/452). *OPA1* is responsible for DOA+, which is a neurologic disorder characterized primarily by the gradual onset of OA and hearing loss during childhood [5], with a wide range of intermediate and variable phenotypes [12]. The clinical presentation of patients in this series is heterogeneous as in other reports on the *OPA1* phenotype. Not all patients with *OPA1* variant exhibited visual impairments, and asymptomatic patients are not rare in DOA [25, 26]. It has been reported that some DOA patients may display only subtle optic-disk atrophy or even a normal-looking optic disk [27]. In our study, 4 individuals carried *OPA1* variants may not exhibit visual impairments (the proband and his mother in Family 1707823, the parents of the proband in Family Y2953973). The severity of both visual impairment and hearing loss, as well as the presence of additional neurological manifestations, exhibit inter- and intra-familial variability. All *OPA1*-related AN in this study exhibited a late-onset age ranging from 20 months to 24 years. We summarized the main clinical phenotypes and onset ages of several reported hereditary forms of AN in Table S2.

Genetic analysis revealed a total of eight variants in *OPA1*. The c.356T >C variant have been reported as a pathogenic variant in a homozygous proband, who exhibited typical OA [28]. The c.1208T >C variant were initially reported in 2015, and it was identified in a 43-year-old man whose early symptoms were like those observed in the proband in this study [29]. The c.1201G >C variant was initially reported in 2014 as part of a study on *OPA1* mutation screening [30]. Although these three variants have been reported, they were all initially reported to association with hearing impairment or confirmed as AN.

Previous studies have shown that missense variants in the GTPase domain were associated with a higher risk of developing the DOA + phenotype [12, 13, 18]. The findings of our study provide evidence to support the observation that all the variants located in the GTPase domain were missense variants. The findings are consistent with previously reported genotype-phenotype correlations, which indicate a 2- to 4-fold increased risk of developing DOA + in individuals with missense mutations and those affecting the GTPase domain [12, 13]. This suggests missense mutations in the GTPase of *OPA1* appear to exert a more potent deleterious impact, potentially through a dominant negative mechanism and an increase in mitochondrial DNA instability [12].

The spectrum of *OPA1*-related disease has expanded to include biallelic inheritance, which has been suggested to result in more severe manifestations in affected individuals [31]. The inheritance of *OPA1* in an AR patten, such as Y2953973, is exceptionally rare. We present here a little girl who carry biallelic *OPA1* variants exhibited a severe phenotype including early-onset progressive visual impairment, nystagmus and retarded development. These two *OPA1* variants c.356T >C and c.2013 + 5G >C from her parents respectively, while her parents were unaffected. The observation of c.356T >C in a Chinese family in another research, along with the presence of homozygous pathogenic variants following an autosomal recessive pattern, is noteworthy [28]. Both parents of the proband in this Chinese family were heterozygous carriers of the c.356T >C variant. They exhibited no clinical symptoms, and their basal/ATP levels were within normal ranges. However, a slight reduction in respiratory reserve capacity was observed, which may indicate potential mitochondrial dysfunction, although not severe enough to manifest as disease [28]. Some researchers have provided genetic and functional evidence that deep intronic mutations and intragenic modifiers in *OPA1* can cause disease [32]. The variant c.356T >C was located in exon 4 and may not cause significant protein functional abnormalities. The degree of mitochondrial damage in cells and the severity of diseases caused by different variants exhibit significant variation [33]. One *OPA1* variants may not cause a phenotype, since the penetrance or mosaic, but acts as a phenotypic modifier if it encounters in trans with another *OPA1* variants. The spectrum of clinical phenotypes associated with *OPA1* mutations, whether heterozygous or in biallelic combinations, may further extend to Behr syndrome [10]. Another transversion variant at the opposite position, c.2013 + 5G >C, was found in this proband in our study. Both of two different splicing prediction software (SpliceAI and MaxEntScan) indicated that this splicing variant would affect the normal splicing of mRNA [34, 35].

OPA1 is involved in multiple functions, including: (1) mediating mitochondrial fusion and organizing the mitochondrial network [36, 37]; (2) association with oxidative phosphorylation and maintenance of membrane potential [38]; (3) organization of cristae and regulation of apoptosis [39, 40]. OPA1 have been localized in both inner and outer hair cells, auditory nerve terminals, and spiral ganglion cells [41, 42]. Therefore, it is not unexpected that disruptions in *OPA1* would impact hearing function. In this study, *OPA1*-related patients all exhibited typical audiological features of AN. The patients noticed a decrease in hearing impairment before the second decades of life and AN manifested as the initial symptom in two patients. The clinical features of patients with AN primarily involves the ability to hear sounds while an inability to understand the meaning. The DPOAE was unsuccessful recorded in the proband from Family 1,707,823, potentially attributed to the gradual attenuation of DPOAE with AN prolongation. Comparatively, CM exhibits higher accuracy than DPOAE [43]. Furthermore, A proband’s natural history (10-year follow-up) demonstrated a progressive deterioration in auditory function, and the annual rates of hearing loss at the frequencies of speech were 2.74 dB/year. Otherwise, followed-up found that a significant decrease in SDS as the disease progressed, which indicated the neurological synchronization of the proband was gradually aggravated. *OPA1* mutations may affect the terminal dendrites of auditory nerve fiber. Cochlear implantation can pass the site of lesion to improve auditory function [18]. However, the current evidence cannot rule out the possibility that *OPA1* mutations may impact IHCs. In rat model, OPA1 expression has been demonstrated in both IHCs and SGNs [41]. Supportively, a mouse model carrying *Opa1* mutations revealed that while auditory nerve fiber terminals first exhibited abnormalities at 1 month of age, IHCs also displayed significant deficits by 6 months of age [42]. Long-term follow-up are warranted to elucidate changes in IHCs.

We performed ECochG in 2 probands, which revealed that -SP wave existed, while CAP wave may be absence. The -SP/AP values were much higher than normal, when -SP and CAP waves existed together. The application of ECochG has been proposed for the diagnosis of AN in order to delineate the specific characteristics of both ribbon synapse and neural responses across different manifestations of this disorder [44]. The -SP waves primarily originate from the IHC, which is an objective indicator of IHC. *OPA1*-linked patients exhibited clearly -SP wave, suggesting that the lesion caused by *OPA1* mutation may be located in the auditory pathway outside the IHC. The production of CAP occurs in SGN and elicits an afferent nerve response. The inability to record CAP and significantly higher -SP/AP values (>0.4) suggested decreased activity, desynchronization, or hypoplasia of auditory nerve. Similar results were observed in the ECochG tests conducted on other *OPA1*-related patients [45, 46]. Because of the characteristics of ECochG, the test results obtained from *OPA1-*related patients were largely consistent. However, in contrast, the PTA was progressively declining. PTA evaluated the ability to detect sound intensity and discriminate frequency. AN involves lesions in the auditory pathway, particularly at the synapses between inner hair cells and spiral ganglion cells, which are critical for encoding temporal auditory information. Temporal processing depends not only on intact auditory function but also on neural integration across auditory nuclei; therefore, the impairment along the auditory pathway may be greater than what is predicted by PTA thresholds. Compared to PTA, EcochG offers greater sensitivity for detecting auditory nerve activity, directly identifying desynchronization, while PTA merely evaluates intensity and frequency detection capabilities [47].

Except cochlear and auditory fiber, OPA1 also expression in the saccule, the utricle, the cristae ampullaris and vestibular neurons of vestibular organ [41]. In this study, gait instability observed in a subset of *OPA1*-related patients suggested potential vestibular dysfunction. Vision impairments and probable muscle-related impairments associated with OPA1 mutations may exacerbate this phenotype. Most critically, abnormal cVEMP responses were detected in these patients, consistent with vestibular impairment previously documented in other reported *OPA1*-related patients [48]. OPA1 deficiency may impair energy-dependent processes such as axoplasmic transport in vestibular nerve fibers, and nerve atrophy in the inner ear may progressively extend from the superior vestibular nerve to the inferior vestibular nerve [48]. In pathological conditions, mitochondrial dysfunction may initiate a vicious cycle whereby oxidative stress and myelin damage are exacerbated [49]. Beyond *OPA1*, vestibular dysfunction is also recognized in *AIFM1*-related AN, with vertigo representing the second most prevalent symptom [50]. Notably, the pathogenic mechanisms diverge from those in *OPA1*-related AN, *AIFM1* mutations impair mitochondrial respiratory chain complex assembly, activating caspase-independent apoptotic pathways [51]. Other AN lack consistent reports of vestibular impairments may arise from variant-specific expression patterns of causative genes, or could reflect masking by severe central nervous system deficits—as exemplified by CAPOS syndrome (caused by *ATP1A3* mutations), where predominant encephalopathic features may obscure vestibular dysfunction [52].

## Conclusion

In summary, the distinct genetic patterns of *OPA1* should be carefully considered in genetic counseling to assess the risk of disease inheritance, and prompt intervention should be implemented for probands due to the potential deterioration of hearing loss. (1) We found seven *OPA1-*related probands (1.55%, 7/452) in AN with eight variants of *OPA1*. All variants in the GTPase domain of *OPA1* were missense variants and most probands (85.71%, 6/7) carried one of missense variants in *OPA1.* (2) Seven probands from seven unrelated families exhibited distinct genetic patterns, including a rare AR inheritance. Proband with AR inheritance exhibited a more severe phenotype. (3) The phenotypic variants among probands are heterogeneous, necessitating a comprehensive clinical assessment and ongoing monitoring to notice hearing deterioration. It is crucial not to overlook the importance of conducting routine examinations on suspected relatives. (4) High-throughput sequencing can be employed for individuals exhibiting atypical phenotypes, aiding in accurate diagnosis.

## Supplementary Information

Below is the link to the electronic supplementary material.

Supplementary Material 1.

## Data Availability

The datasets generated in this study have been included in the article. For inquiries or requests to access the data, please contact prof. Qiuju Wang (wqjavm301@sina.com).

## Abbreviations

ABR: Auditory Brainstem Responses
ACMG/AMP: American College of Medical Genetics And Genomics/ Association For Molecular Pathology
AD: Autosomal Dominant
AN: Auditory Neuropathy
AR: Autosomal Recessive
CAP: Compound Action Potential
CM: Cochlear Microphonics
CoAP: Categories of Auditory Performance
CT: Computed Tomography
cVEMP: Cervical Vestibular Evoked Myogenic Potentials
DOA: Autosomal Dominant Optic Atrophy
ECochG: Electrocochleogram
FVEP: Flash Visual Evoked Potential
HHL: Hereditary Hearing Loss
IHCs: Inner Hair Cell
MRI: Magnetic Resonance Imaging
MCV: Motor Nerve Conduction Velocity
OA: Optic Neuropathy
OAE: Otoacoustic Emission
OCT: Optical Coherence Tomography
OHC: Outer Hair Cell
PERG: Pattern Electroretinogram
PTA: Pure Tone Audiometry
REVEL: Rare Exome Variant Ensemble Learner
SDS: Speech Discrimination Score
SGNs: Spiral Ganglion Neuron
SIFT: Sorting Intolerant from Tolerant
SIR: Speech Intelligibility Rating
SNAP: Sensory Nerve Action Potential
SP/AP: Summating Potentials/ Action Potential
WGS: Whole Genome Sequencing
