# Supplementary material for: Distinct genetic patterns and natural history of OPA1-related auditory neuropathy in Chinese population
Source: Orphanet J Rare Dis. 2025 Oct 17;20:520. doi: 10.1186/s13023-025-04040-4 (PMC12534986; doi:10.1186/s13023-025-04040-4)
Supplement: Supplementary file 1 — Supplementary Material 1. [file 13023_2025_4040_MOESM1_ESM.docx]

**Table S1 Reported Variations of *OPA1*-Related Patients with Hearing Loss**

| Nation | | Nucleic acid change  (NM_ 015560.3) | Amino acid change | Main clinical features | Reference |
| --- | --- | --- | --- | --- | --- |
| 1 | England | c.113_130del | p.Arg38_Ser43del | SNHL, myopathy, PEO | [1] |
| 2 | Japan | c.154C>T | p.Arg52* | OA, SNHL | [2] |
| 3 | China | c.356T>C | p.Phe119Ser | OA, AN, nystagmus, strabismus, retarded development | This Study |
| 4 | England | c.635_636del | p.Lys212fs*4 | OA, SNHL | [1] |
| 5 | England, France | c.728T>A | p.Leu243* | OA, SNHL, ataxia, neuropathy, paraparesis, posterior cord syndrome | [1, 3] |
| 6 | China | c.805T>C | p.Ser269Pro | OA, AN, nystagmus, gait instability | This Study |
| 7 | Australian | c.888T>A | p.Asp296Glu | OA, SNHL, ataxic, ptosis, ophthalmoplegia, proximal myopathy, neuropathy and ataxia | [4] |
| 8 | Japan | c.892A>C | p.Ser298Arg | OA, AN | [5] |
| 9 | England | c.970_978del | p.Arg324_Pro326del | OA, SNHL | [1] |
| 10 | France | c.983A>G | p.Val291_Phe328del | OA, SNHL | [3] |
| 11 | England | c.1065+1G>A | - | OA, SNHL, multiple sclerosis-like | [6] |
| 12 | France | c.1067_1068dup | p.Ala357Leufs*4 | OA, SNHL | [3] |
| 13 | France | c.1069G>A | p.Ala357Thr | OA, SNHL, ptosis, ophthalmoplegia, ataxia, myopathy, sensory-motor axonal neuropathy | [7] |
| 14 | England | c.1198C>T | p.Pro400Ser | OA, SNHL, PEO | [1] |
| 15 | China | c.1201G>C | p.Gly401Arg | OA, AN | This Study |
| 16 | China, England, France | c.1202G>A | p.Gly401Asp | OA, SNHL, ataxia, paraesthesia, nystagmus, peripheral neuropathy | [1, 3, 8] |
| 17 | China | c.1208T>C | p.Ile403Thr | OA, AN, trichiasis, gait instability | This Study |
| 18 | England | c.1212+3A>T | - | OA, SNHL | [1] |
| 19 | Japan | c.1240A>C | p.Thr414Pro | OA, AN | [9] |
| 20 | China | c.1303T>C | p.Cys435Arg | OA (poor vision and optic-disk pallor), SNHL, nystagmus | [10] |
| 21 | China | c.1304G>A | p.Cys435Tyr | OA (optic disc pallor and diffused RNFL defects), SNHL | [11] |
| 22 | France | c.1310A>G | p.Gln437Arg | OA, SNHL, ataxia, paraesthesia, nystagmus | [3] |
| continued | | | | | |
| 23 | England | c.1313A>T | p.Asp438Val | OA, SNHL | [1] |
| 24 | Italy, England | c.1316G>T | p.Gly439Val | OA, SNHL, ataxic, ophthalmoplegia, sensory-motor axonal neuropathy, myopathy, PEO, neuropathy | [1, 7, 12] |
| 25 | England | c.1327G>A | p.Ala443Thr | OA, SNHL, ataxia, CAPOS | [1] |
| 26 | China | c.1333C>G | p.Arg445Gly | OA, AN | This Study |
| 27 | France, Japan, USA, France, Spain, China | c.1334G>A | p.Arg445His | OA (temporal pallor of the disc, with parapapillary RNFL defect), AN, ptosis, ophthalmoplegia, myopathy, neuropathy, PEO, Dementia, sensorimotor neuropathy, muscle pain and fatigue in both lower limbs after long walks, vestibular dysfunction | [1, 3, 5, 7, 11, 13-17] |
| 28 | Czech Republic | c.1345A>C | p.Thr449Pro | OA, SNHL, ataxia, neuropathy, migraine, tachycardia, intention tremor, nystagmus, cervical dystonia | [18] |
| 29 | France | c.1387_1392dup | p.Ile463_Phe464dup | OA, SNHL, nystagmus, ophthalmoplegia, axonal sensorimotor neuropathy | [3] |
| 30 | England | c.1462A>G | p.Gly488Arg | OA, SNHL, ataxia, myopathy, neuropathy, PEO, Migraine, epilepsy, Dementia | [1] |
| 31 | England | c.1484C>T | p.Ala495Val | OA, SNHL, myopathy, neuropathy, PEO | [1] |
| 32 | China | c.1555G>A | p.Glu519Lys | OA, AN | This Study |
| 33 | Japan | c.1618A>C | p.Thr540Pro | OA, AN | [5, 9] |
| 34 | France, England | c.1635C>G | p.Ser545Arg | OA, SNHL, ptosis, ataxia, ophthalmoplegia, ataxia, myopathy, sensory motor axonal neuropathy, PEO | [1, 7, 19] |
| 35 | England | c.1642G>A | p.Val548Ile | OA, SNHL, ataxia | [1] |
| 36 | England | c.1745A>G | p.Tyr582Cys | OA, SNHL, ataxia, PEO | [1] |
| 37 | China | c.2013+5G>C | - | OA, AN, nystagmus, strabismus, retarded development | This Study |
| continued | | | | | |
| 38 | China | c.2635_2639del | p.Leu879Leufs*22 | OA, AN | This Study |
| 39 | England | c.2800dup | p.Gln934Profs*10 | OA, SNHL, peripheral sensitive axonal neuropathy | [6] |
| 40 | China | c.2844dup | p.Leu949Thrfs*2 | OA (poor vision and optic-disk pallor), SNHL | [10] |
| 41 | China, England | c.2848_2849del | p.Asp950Cysfs*4 | OA (progressive decrease in visual acuity, color vision abnormalities, thinned optic nerves, and moderate to severe temporal optic nerve pallor), SNHL, high myopia, ataxia | [1, 2] |

Note: AN: auditory neuropathy; OA: optic atrophy; SNHL: sensorineural hearing loss; PEO: progressive external ophthalmoplegia

Table S2 Summary of Clinical Characteristics of Genetic Auditory Neuropathy

| **AN gene** | **Onset age** | **Major clinical features** | **Clinical progression** | **Reference** |
| --- | --- | --- | --- | --- |
| *OPA1* | 20 months – 24 years | AN, OA | progression | This Study |
| *OTOF* | Congenital | AN | stable | [20] |
| *AIFM1* | 5 years – 20 years | AN, peripheral neuropathy | progression | [21] |
| *FDXR* | 10 years and 16 years | AN, peripheral neuropathy | progression | [22] |
| *TWNK* | 2 years – 25 years | AN, peripheral neuropathy | progression | [22] |
| *ATP1A3* | 2 years – 25 years | AN, CAPOS | progression | [23] |
| *TIMM8A* | first decade of life | AN, MTS | progression | [24] |
| *DIAPH1* | 8 years – 49 years | AN | progression | [25] |

Note: AN: auditory neuropathy; OA: optic atrophy; CAPOS: Cerebellar ataxia, areflexia, pes cavus, optic atrophy, and sensorineural hearing loss; MTS: Mohr-Tranebjaerg syndrome.

**Reference**

1. Yu-Wai-Man P, Griffiths PG, Gorman GS, Lourenco CM, Wright AF, Auer-Grumbach M, Toscano A, Musumeci O, Valentino ML, Caporali L *et al*: **Multi-system neurological disease is common in patients with OPA1 mutations**. *Brain : a journal of neurology* 2010, **133**(Pt 3):771-786.

2. Ban Y, Yoshida Y, Kawasaki S, Mochida C: **A novel mutation of the OPA1 gene in a Japanese patient with autosomal dominant optic atrophy**. *Graefes Arch Clin Exp Ophthalmol* 2007, **245**(10):1581-1583.

3. Leruez S, Milea D, Defoort-Dhellemmes S, Colin E, Crochet M, Procaccio V, Ferré M, Lamblin J, Drouin V, Vincent-Delorme C *et al*: **Sensorineural hearing loss in OPA1-linked disorders**. *Brain : a journal of neurology* 2013, **136**(Pt 7):e236.

4. Ahmad KE, Davis RL, Sue CM: **A novel OPA1 mutation causing variable age of onset autosomal dominant optic atrophy plus in an Australian family**. *J Neurol* 2015, **262**(10):2323-2328.

5. Maeda-Katahira A, Nakamura N, Hayashi T, Katagiri S, Shimizu S, Ohde H, Matsunaga T, Kaga K, Nakano T, Kameya S *et al*: **Autosomal dominant optic atrophy with OPA1 gene mutations accompanied by auditory neuropathy and other systemic complications in a Japanese cohort**. *Molecular vision* 2019, **25**:559-573.

6. Liskova P, Tesarova M, Dudakova L, Svecova S, Kolarova H, Honzik T, Seto S, Votruba M: **OPA1 analysis in an international series of probands with bilateral optic atrophy**. *Acta Ophthalmol* 2017, **95**(4):363-369.

7. Amati-Bonneau P, Valentino ML, Reynier P, Gallardo ME, Bornstein B, Boissière A, Campos Y, Rivera H, de la Aleja JG, Carroccia R *et al*: **OPA1 mutations induce mitochondrial DNA instability and optic atrophy 'plus' phenotypes**. *Brain : a journal of neurology* 2008, **131**(Pt 2):338-351.

8. Ke T, Nie SW, Yang QB, Liu JP, Zhou LN, Ren X, Liu JY, Wang Q, Liu MG: **The G401D mutation of OPA1 causes autosomal dominant optic atrophy and hearing loss in a Chinese family**. *Zhonghua yi xue yi chuan xue za zhi = Zhonghua yixue yichuanxue zazhi = Chinese journal of medical genetics* 2006, **23**(5):481-485.

9. Namba K, Mutai H, Takiguchi Y, Yagi H, Okuyama T, Oba S, Yamagishi R, Kaneko H, Shintani T, Kaga K *et al*: **Molecular Impairment Mechanisms of Novel OPA1 Mutations Predicted by Molecular Modeling in Patients With Autosomal Dominant Optic Atrophy and Auditory Neuropathy Spectrum Disorder**. *Otology & neurotology : official publication of the American Otological Society, American Neurotology Society [and] European Academy of Otology and Neurotology* 2016, **37**(4):394-402.

10. Li H, Jones EM, Li H, Yang L, Sun Z, Yuan Z, Chen R, Dong F, Sui R: **Clinical and genetic features of eight Chinese autosomal-dominant optic atrophy pedigrees with six novel OPA1 pathogenic variants**. *Ophthalmic genetics* 2018, **39**(5):569-576.

11. Chen J, Xu K, Zhang X, Jiang F, Liu L, Dong B, Ren Y, Li Y: **Mutation screening of mitochondrial DNA as well as OPA1 and OPA3 in a Chinese cohort with suspected hereditary optic atrophy**. *Investigative ophthalmology & visual science* 2014, **55**(10):6987-6995.

12. Liguori M, La Russa A, Manna I, Andreoli V, Caracciolo M, Spadafora P, Cittadella R, Quattrone A: **A phenotypic variation of dominant optic atrophy and deafness (ADOAD) due to a novel OPA1 mutation**. *J Neurol* 2008, **255**(1):127-129.

13. Amati-Bonneau P, Odent S, Derrien C, Pasquier L, Malthiéry Y, Reynier P, Bonneau D: **The association of autosomal dominant optic atrophy and moderate deafness may be due to the R445H mutation in the OPA1 gene**. *American journal of ophthalmology* 2003, **136**(6):1170-1171.

14. Shimizu S, Mori N, Kishi M, Sugata H, Tsuda A, Kubota N: **A novel mutation in the OPA1 gene in a Japanese patient with optic atrophy**. *American journal of ophthalmology* 2003, **135**(2):256-257.

15. Payne M, Yang Z, Katz BJ, Warner JE, Weight CJ, Zhao Y, Pearson ED, Treft RL, Hillman T, Kennedy RJ *et al*: **Dominant optic atrophy, sensorineural hearing loss, ptosis, and ophthalmoplegia: a syndrome caused by a missense mutation in OPA1**. *American journal of ophthalmology* 2004, **138**(5):749-755.

16. Li C, Kosmorsky G, Zhang K, Katz BJ, Ge J, Traboulsi EI: **Optic atrophy and sensorineural hearing loss in a family caused by an R445H OPA1 mutation**. *American journal of medical genetics Part A* 2005, **138a**(3):208-211.

17. Amati-Bonneau P, Guichet A, Olichon A, Chevrollier A, Viala F, Miot S, Ayuso C, Odent S, Arrouet C, Verny C *et al*: **OPA1 R445H mutation in optic atrophy associated with sensorineural deafness**. *Annals of neurology* 2005, **58**(6):958-963.

18. Liskova P, Ulmanova O, Tesina P, Melsova H, Diblik P, Hansikova H, Tesarova M, Votruba M: **Novel OPA1 missense mutation in a family with optic atrophy and severe widespread neurological disorder**. *Acta Ophthalmol* 2013, **91**(3):e225-e231.

19. Hudson G, Amati-Bonneau P, Blakely EL, Stewart JD, He L, Schaefer AM, Griffiths PG, Ahlqvist K, Suomalainen A, Reynier P *et al*: **Mutation of OPA1 causes dominant optic atrophy with external ophthalmoplegia, ataxia, deafness and multiple mitochondrial DNA deletions: a novel disorder of mtDNA maintenance**. *Brain : a journal of neurology* 2008, **131**(Pt 2):329-337.

20. Zhang QJ, Han B, Lan L, Zong L, Shi W, Wang HY, Xie LY, Wang H, Zhao C, Zhang C *et al*: **High frequency of OTOF mutations in Chinese infants with congenital auditory neuropathy spectrum disorder**. *Clin Genet* 2016, **90**(3):238-246.

21. Wang H, Bing D, Li J, Xie L, Xiong F, Lan L, Wang D, Guan J, Wang Q: **High Frequency of AIFM1 Variants and Phenotype Progression of Auditory Neuropathy in a Chinese Population**. *Neural Plast* 2020, **2020**:5625768.

22. Cao Y, Zhang X, Lan L, Li D, Li J, Xie L, Xiong F, Yu L, Wu X, Wang H *et al*: **Identification of genetic mechanisms of non-isolated auditory neuropathy with various phenotypes in Chinese families**. *Orphanet J Rare Dis* 2025, **20**(1):11.

23. Wang W, Li J, Lan L, Xie L, Xiong F, Guan J, Wang H, Wang Q: **Auditory Neuropathy as the Initial Phenotype for Patients With ATP1A3 c.2452 G > A: Genotype-Phenotype Study and CI Management**. *Frontiers in cell and developmental biology* 2021, **9**:749484.

24. Wang H, Wang L, Yang J, Yin L, Lan L, Li J, Zhang Q, Wang D, Guan J, Wang Q: **Phenotype prediction of Mohr-Tranebjaerg syndrome (MTS) by genetic analysis and initial auditory neuropathy**. *BMC medical genetics* 2019, **20**(1):11.

25. Wu K, Wang H, Guan J, Lan L, Zhao C, Zhang M, Wang D, Wang Q: **A novel variant in diaphanous homolog 1 (DIAPH1) as the cause of auditory neuropathy in a Chinese family**. *International journal of pediatric otorhinolaryngology* 2020, **133**:109947.
